# Incidence and Characterization of Post-COVID-19 Symptoms in Hospitalized COVID-19 Survivors to Recognize Syndemic Connotations in India: Single-Center Prospective Observational Cohort Study

**DOI:** 10.2196/40028

**Published:** 2023-04-18

**Authors:** Chithira V Nair, Merlin Moni, Fabia Edathadathil, Appukuttan A, Preetha Prasanna, Roshni Pushpa Raghavan, Dipu T Sathyapalan, Aveek Jayant

**Affiliations:** 1 Division of Infectious Diseases Department of General Medicine Amrita Institute of Medical Science and Research Centre Kochi India; 2 Department of Infection Control and Epidemiology Amrita Institute of Medical Science and Research Centre Kochi India; 3 Department of Medical Administration Amrita Institute of Medical Science and Research Centre Kochi India; 4 Department of Pharmacy Practice Amrita School of Pharmacy Kochi India; 5 Department of Anaesthesia, Critical Care and Pain Homi Bhabha Cancer Hospital and Research Centre Visakhapatnam India

**Keywords:** COVID-19, follow-up, incidence, fatigue, long COVID, post-COVID, post-COVID-19 symptoms, questionnaire, tertiary-care center, intensive care, symptom monitoring, prospective observational study, treatment, steroid, viral therapy, postdischarge

## Abstract

**Background:**

Long COVID, or post-COVID-19 syndrome, is the persistence of signs and symptoms that develop during or after COVID-19 infection for more than 12 weeks and are not explained by an alternative diagnosis. In spite of health care recouping to prepandemic states, the post-COVID-19 state tends to be less recognized from low- and middle-income country settings and holistic therapeutic protocols do not exist. Owing to the syndemic nature of COVID-19, it is important to characterize post-COVID-19 syndrome.

**Objective:**

We aimed to determine the incidence of post-COVID-19 symptoms in a cohort of inpatients who recovered from COVID-19 from February to July 2021 at a tertiary-care center in South India. In addition, we aimed at comparing the prevalence of post-COVID-19 manifestations in intensive care unit (ICU) and non-ICU patients, assessing the persistence, severity, and characteristics of post-COVID-19 manifestations, and elucidating the risk factors associated with the presence of post-COVID-19 manifestations.

**Methods:**

A total of 120 adult patients admitted with COVID-19 in the specified time frame were recruited into the study after providing informed written consent. The cohort included 50 patients requiring intensive care and 70 patients without intensive care. The follow-up was conducted on the second and sixth weeks after discharge with a structured questionnaire. The questionnaire was filled in by the patient/family member of the patient during their visit to the hospital for follow-up at 2 weeks and through telephone follow-up at 6 weeks.

**Results:**

The mean age of the cohort was 55 years and 55% were men. Only 5% of the cohort had taken the first dose of COVID-19 vaccination. Among the 120 patients, 58.3% had mild COVID-19 and 41.7% had moderate to severe COVID-19 infection. In addition, 60.8% (n=73) of patients had at least one persistent symptom at the sixth week of discharge and 50 (41.7%) patients required intensive care during their inpatient stay. The presence of persistent symptoms at 6 weeks was not associated with severity of illness, age, or requirement for intensive care. Fatigue was the most common reported persistent symptom with a prevalence of 55.8%, followed by dyspnea (20%) and weight loss (16.7%). Female sex (odds ratio [OR] 2.4, 95% CI 1.03-5.58; *P*=.04) and steroid administration during hospital stay (OR 4.43, 95% CI 1.9-10.28; *P*=.001) were found to be significant risk factors for the presence of post-COVID-19 symptoms at 6 weeks as revealed by logistic regression analysis.

**Conclusions:**

Overall, 60.8% of inpatients treated for COVID-19 had post-COVID-19 symptoms at 6 weeks postdischarge from the hospital. The incidence of post-COVID-19 syndrome in the cohort did not significantly differ across the mild, moderate, and severe COVID-19 severity categories. Female sex and steroid administration during the hospital stay were identified as predictors of the persistence of post-COVID-19 symptoms at 6 weeks.

## Introduction

The COVID-19 pandemic has posed unprecedented challenges to the hubris of modern medicine. Even as the understanding of viral structure, its variants, transmission, disease biology, and other factors evolves, the substantial aftermath of infection in the form of long COVID, or post-COVID-19 syndrome, could pose lingering challenges [1]. Presentations of multisystem inflammatory syndrome in children and adults show that COVID-19 negativity is not a hardtop, as there could be postinfectious complications constituting a post-COVID-19 state and its associated health issues [2].

A clear definition of what these health problems are and their magnitude is crucial to prepare an efficient, multidisciplinary service [3]. The World Health Organization has highlighted that less than 1% of the total pandemic literature has focused on the post-COVID-19 condition [4]. The pandemic has also spurred tremendous social and economic upheaval across society; lingering post-COVID-19 health problems markedly magnify this stress at both the individual and community levels [5]. This situation leads to the wider implication that the COVID-19 pandemic has been, in the truest sense, a syndemic for southern Asia. A syndemic is not merely a comorbidity but is rather characterized by biological and social interactions between conditions and states, including interactions that increase a person’s susceptibility to harm or worsen their health outcomes, representing a facet of health care that although is well known is also famously ignored [6].

Long COVID, or post-COVID-19 syndrome, has been defined as signs and symptoms that develop during or after an infection consistent with COVID-19, continue for more than 12 weeks, and are not explained by an alternative diagnosis [7]. The first fundamental realization of this syndrome has been that these symptoms could arise in patients across the severity spectrum of the infection, including those without any symptoms [8]. Post-COVID-19 symptoms can range from fatigue, myalgia, dyspnea, dry cough, and loss of smell and taste to thrombotic complications, among which fatigue seems to be the most common presentation [9,10]. The frequency with which these complications occur or persist seems to drop off with time beyond the acute phase [11]. As a dynamic disease entity with newer variants and the vaccines changing the disease biology, including that of post-COVID-19 syndrome, it is important to characterize the symptoms at different time points in the pandemic and also in patients with varying disease severity to gain a better understanding of the full spectrum of this new disease entity.

In spite of health care recouping to prepandemic states, the post-COVID-19 state tends to be less recognized and holistic therapeutic protocols do not exist [12]. On the ground, it is also often necessary to recognize and treat residual organ damage such as lung fibrosis or thromboembolic sequelae differently from the fatigue-like state that is common following many infections [13,14]. Although various therapeutic options are being explored on a clinical trial basis, including anti-inflammatory medications, lack of data, especially from low- and middle-income country (LMIC) settings, represents a huge drawback with regard to identification of the specific population requiring medical attention after contracting this viral illness [15,16]. In addition, various other realms of health, such as cognitive loss, psychiatric illness, nutrition, and sleep hygiene, need to be embedded in the clinical care pathway [17,18]. Moreover, recognition of patient- and disease-specific aspects that might predispose an individual to contracting post-COVID-19 syndrome is necessary.

In this backdrop, the aim of this prospective study was to determine the incidence of post-COVID-19 syndrome in a cohort of patients hospitalized for COVID-19 during the time period of February to July 2021 from a tertiary-care hospital in South India at 6 weeks postdischarge of inpatient stay. In addition, we aimed at comparing the prevalence of post-COVID-19 manifestations in intensive care unit (ICU) and non-ICU patients, assessing the persistence, severity, and characteristics of post-COVID-19 manifestations, and elucidating the risk factors associated with the presence of post-COVID-19 manifestations.

## Methods

### Study Setting

This was a single-center, hospital-based prospective observational study conducted at a 1300-bed academic tertiary-care referral center in South India from February to July 2021. The hospital had a dedicated isolation facility comprising ICU and non-ICU locations for treating patients admitted with COVID-19. Eligible patients included those aged ≥18 years and were hospitalized in a COVID-19 isolation unit for COVID-19 based on a positive SARS-CoV-2 reverse transcription-polymerase chain reaction or antigen test at the time of admission.

**Figure 1:**
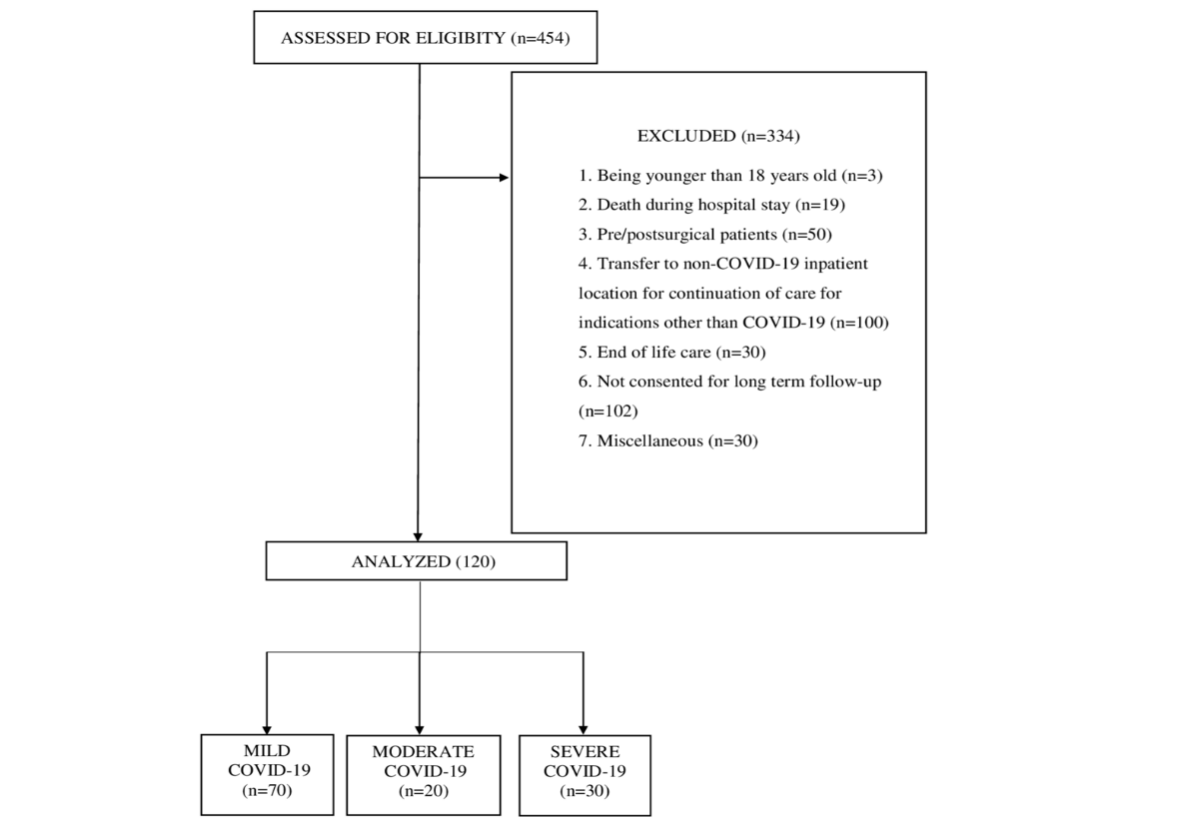
Patient selection for this prospective cohort study.

All patients who were admitted to the COVID-19 isolation unit during the specified period were screened for eligibility and those meeting eligibility criteria were recruited after providing informed written consent. Patients younger than 18 years of age, those with a life condition triggering palliative intent, those with COVID-19 and a surgical indication (intended or actual), or those transferred outside specific COVID-19 wards for continued care for conditions not directly triggered by the COVID-19 infection were excluded. Patients who did not consent to long-term follow-up postdischarge were also excluded from the study (Figure 1).

### In-Hospital Data Collection

The data collection included demographic characteristics such as age, sex, social history, and comorbidities. We also recorded COVID-19–specific parameters, including the severity of COVID-19 infection, level of clinical care received (ICU or non-ICU locations), length of hospital stay (LOS), vaccination status, disease symptoms, and treatment details (eg, antiviral, anticoagulation, and steroid administration), in COVID-19 patients.

### Follow-up After Discharge

A questionnaire was prepared to assess the post-COVID-19 symptoms in patients after their discharge from the hospital. The follow-up was conducted at 2 weeks (first follow-up) and 6 weeks (second follow-up) after discharge. The post-COVID-19 symptoms were grouped into “acute COVID-19” and “ongoing symptomatic COVID-19” categories based on the 2-week and 6-week timeline postdischarge, respectively [7]. Information regarding the current symptoms and working status of each patient was obtained. Face-to-face interviews and questionnaire completion were performed during the patients’ hospital visit for follow-up. Patients who were not able to come to the hospital were followed up by telephone. The collected data were entered in a predesigned data collection form and populated in a Microsoft Excel sheet for further analysis.

### Statistical Analysis

Statistical analysis was performed using IBM SPSS version 20.0 software. Categorical variables are summarized using frequency and percentage. Numerical variables are presented using mean (SD). To test the statistical significance of the comparison of categorical variables between two subject groups, a *χ^2^* test with continuity correction was applied. To test the statistical significance of the difference in the mean values of numerical variables between two subject groups, the Student *t*-test was used. Logistic regression was employed to identify the predictors of presence of symptoms lingering at 6 weeks. *P*<.05 was considered to indicate a statistically significant difference.

### Ethical Considerations

The study participants were informed that participation was anonymous and voluntary, that all responses would be kept confidential, and that the collected data would be used for academic research only. The study was approved by the Institutional Review Board of Amrita Institute of Medical Sciences and Research Centre (approval number IEC-AIMS-2021-PHARM-095), and all participants provided written informed consent.

## Results

Among the 454 patients who were screened and assessed for eligibility, 334 patients were excluded (Figure 1). Thus, a total of 120 patients were involved in the study, with 70 (58.3%) patients receiving clinical care at non-ICU (mild disease) locations and 50 (41.7%) patients receiving care at ICU locations, including 20 (16.7%) with moderate disease and 30 (25.0%) with severe disease. Demographic details and infection-specific characteristics of the patients are depicted in Table 1. The mean age of the study patients was 54.95 (SD 17.5) years and the majority were men. The mean LOS was 16.08 (SD 10.1) days.

**Table 1:** Demographic data and disease characteristics of patients hospitalized for COVID-19 infection.

Variables		Total (N=120)	Non-ICU^a^ patients (n=70)	ICU patients (n=50)
Age (years), mean (SD)		54.95 (17.5)	51.79 (19.7)	59.38 (13.2)
**Age group (years), n (%)**				
	<40	27 (22.5)	22 (31.4)	5 (10.0)
	40-60	47 (39.2)	24 (34.3)	23 (46.0)
	>60	46 (38.3)	24 (34.3)	22 (44.0)
**Sex, n (%)**				
	Male	66 (55.0)	30 (42.9)	36 (72.0)
	Female	54 (45.0)	40 (57.1)	14 (28.0)
**Social history, n (%)**				
	Alcohol consumption	13 (10.8)	6 (8.6)	7 (14.0)
	Smoking	14 (11.7)	7 (10.0)	7 (14.0)
Length of hospital stay (days), mean (SD)		16.08 (10.1)	9.26 (2.7)	25.64 (8.9)
**Charlson Comorbidity Index, n (%)**				
	Mild (1-2)	31 (25.8)	18 (25.7)	13 (26.0)
	Moderate (3-4)	31 (25.8)	14 (20.0)	17 (34.0)
	Severe (≥5)	17 (14.2)	10 (14.3)	7 (14.0)
New onset of diabetes, n (%)		6 (5.0)	4 (5.7)	2 (4.0)
Oxygen requirement, n (%)		49 (40.8)	1 (1.4)	48 (96.0)
**Vaccination status, n (%)**				
	First dose	6 (5.0)	6 (8.6)	0 (0)
	Second dose	0 (0)	0 (0)	0 (0)
**Symptoms at admission, n (%)**				
	Respiratory	62 (51.7)	27 (38.6)	35 (70.0)
	Neuropsychiatric	23 (19.2)	14 (20)	9 (18.0)
	Musculoskeletal	26 (21.7)	17 (24.3)	9 (18.0)
	Cardiovascular	2 (1.7)	2 (2.9)	0 (0)
	Constitutional	73 (60.8)	36 (51.4)	37 (74.0)
	Gastrointestinal	17 (14.2)	9 (12.9)	8 (16.0)

^a^ICU: intensive care unit.

The commonly observed comorbidities in the study cohort were hypertension (39.1%) and diabetes mellitus (39.2%), and 5% of the patients were diagnosed with new onset of diabetes. None of the patients had taken the two-dose primary COVID-19 vaccine before contracting the disease; 6 patients (5.0%) had taken the first dose of the Covishield vaccine. Most of the patients exhibited constitutional symptoms (60.8%) and respiratory symptoms (51.7%) during their hospital stay.

During the second week, 78.3% (94/120) of patients remained symptomatic with at least one listed symptom among fatigue, dyspnea, cough, weight loss, sleep disturbance, and loss of appetite (Table 2).

During the sixth week, 60.8% (73/120) of patients remained symptomatic. At 6 weeks of follow-up, fatigue was the most commonly reported symptom, followed by dyspnea, weight loss, loss of appetite, and cough (Table 2). Anxiety/depression was reported in 8.3% and 5.0% of patients during the 2nd and 6th week of follow-up, respectively. None of the patients had a fever at the 6-week follow-up.

**Figure 2:**
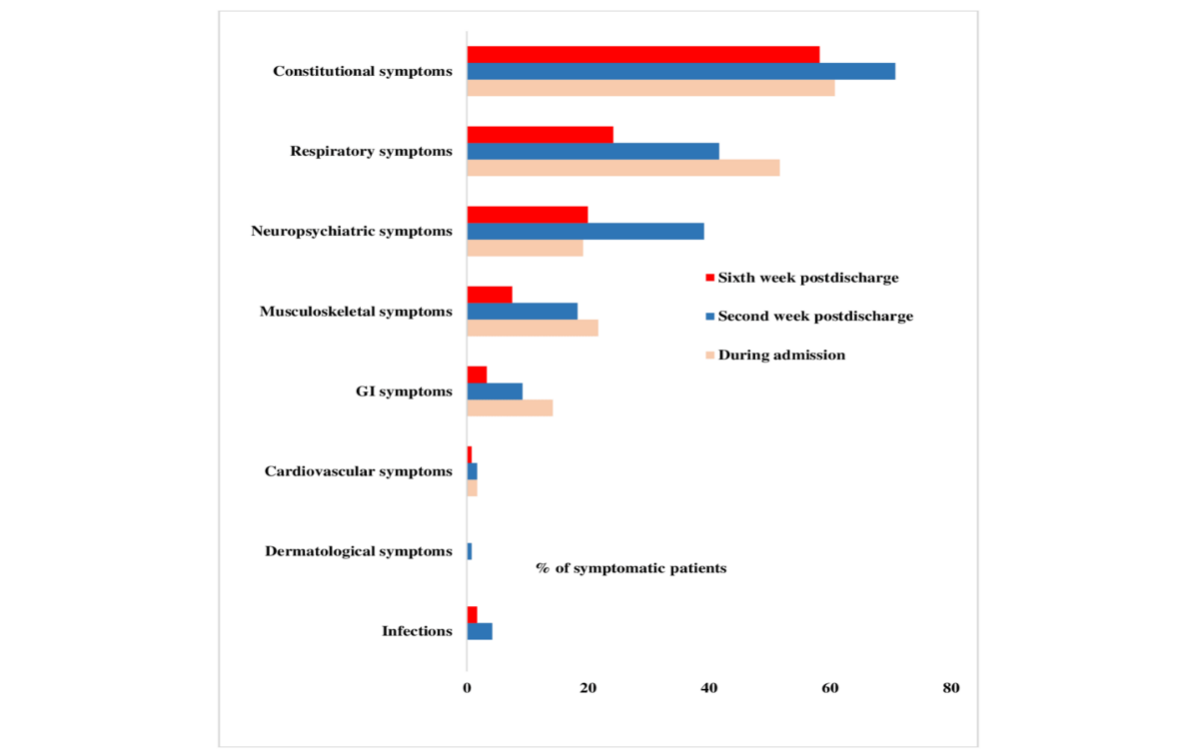
Percentage of patients with COVID-19–related symptoms during hospital admission and at the time of follow-ups. GI: gastrointestinal.

**Figure 3:**
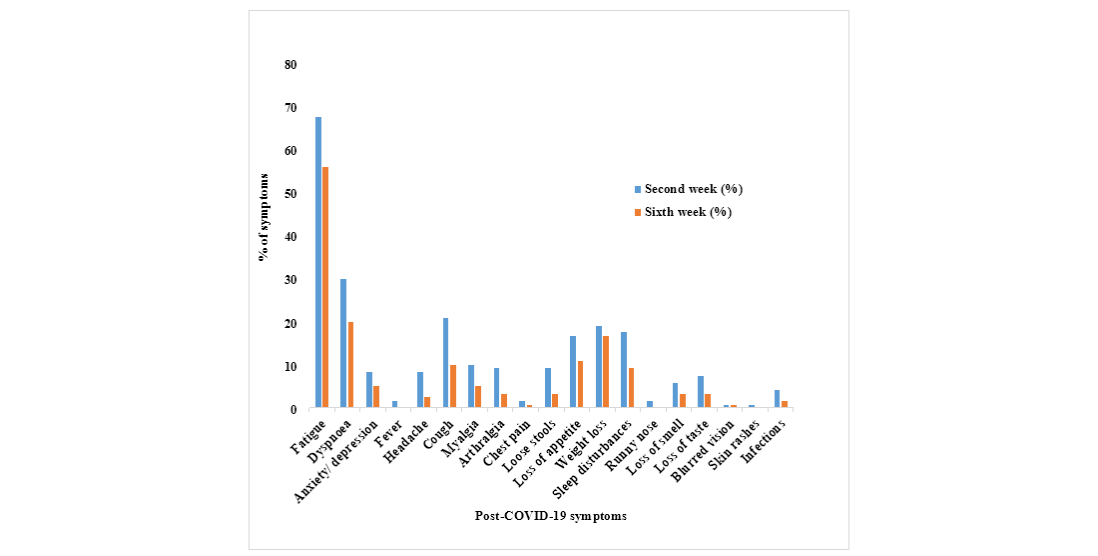
Post-COVID-19 symptoms during the second and sixth week after discharge from the hospital.

The distribution of systemic symptoms of patients with COVID-19 during hospital admission and the presence of post-COVID-19 symptoms at each follow-up are depicted in Figure 2. The change in the post-COVID-19 symptom profile is depicted in Figure 3. The symptoms were classified as constitutional symptoms, including fatigue, fever, sore throat, loss of appetite, and weight loss; respiratory symptoms, including dyspnea, cough, and runny nose; neuropsychiatric symptoms, including headache, anxiety/depression, sleep disturbances, loss of smell, loss of taste, and blurred vision; musculoskeletal symptoms, including myalgia and arthralgia; gastrointestinal symptoms, including loose stools and nausea/vomiting; cardiovascular symptoms, including chest pain and palpitation; dermatological symptoms, including skin rashes; and infections, including lower respiratory tract, urinary tract, and gastrointestinal infections.

**Table 2:** Characterization of postdischarge persistent symptoms during the second and sixth weeks of follow-up.

Symptoms	Week-2 follow-up					Week-6 follow-up			
	Overall (N=120), n (%)	Non-ICU^a^ patients (n=70), n (%)	ICU patients (n=50), n (%)	*P* value	Overall (N=120), n (%)		Non-ICU patients (n=70), n (%)	ICU patients (n=50), n (%)	*P* value
Fatigue	81 (67.5)	44 (62.9)	37 (74.0)	.12	67 (55.8)		38 (54.3)	29 (58.0)	.51
Dyspnea	36 (30.0)	17 (24.3)	19 (38.0)	.11	24 (20.0)		11 (15.7)	13 (26.0)	.17
Anxiety/ depression	10 (8.3)	5 (7.1)	5 (10.0)	.58	6 (5.0)		3 (4.3)	3 (6.0)	>.99
Fever	2 (1.7)	1 (1.4)	1 (2.0)	.99	0 (0)		0 (0)	0 (0)	N/A^b^
Headache	10 (8.3)	5 (7.1)	5 (10.0)	.58	3 (2.5)		1 (1.4)	2 (4.0)	.77
Cough	25 (20.8)	10 (14.3)	15 (30.0)	.04	12 (10.0)		5 (7.1)	7 (14.0)	.22
Myalgia	12 (10.0)	6 (8.6)	6 (12.0)	.54	6 (5.0)		3 (4.3)	3 (6.0)	>.99
Arthralgia	11 (9.2)	5 (7.1)	6 (12.0)	.36	4 (3.3)		2 (2.9)	2 (4.0)	>.99
Chest pain	2 (1.7)	1 (1.4)	1 (2.0)	.99	1 (0.8)		0 (0)	1 (2.0)	.87
Loose stools	11 (9.2)	6 (8.6)	5 (10.0)	.79	4 (3.3)		2 (2.9)	2 (4.0)	>.99
Loss of appetite	20 (16.7)	9 (12.9)	11 (22.0)	.19	13 (10.8)		7 (10.0)	6 (12.0)	.73
Weight loss	23 (19.1)	7 (10.0)	16 (32.0)	.003	20 (16.7)		13 (18.6)	7 (14.0)	.22
Sleep disturbances	21 (17.5)	9 (12.9)	12 (24.0)	.11	11 (9.2)		5 (7.1)	6 (12.0)	.36
Runny nose	2 (1.7)	1 (1.4)	1 (2.0)	.99	0 (0)		0 (0)	0 (0)	N/A
Loss of smell	7 (5.8)	4 (5.7)	3 (6.0)	>.99	4 (3.3)		2 (2.9)	2 (4.0)	>.99
Loss of taste	9 (7.5)	5 (7.1)	4 (8.0)	.99	4 (3.3)		2 (2.9)	2 (4%)	>.99
Blurred vision	1 (0.8)	1 (1.4)	0 (0)	>.99	1 (0.8)		1 (1.4)	0 (0)	>.99
Skin rashes	1 (0.8)	0 (0)	1 (2.0)	.87	0 (0)		0 (0)	0 (0)	N/A
Infections	5 (4.2)	1 (1.4)^c^	4 (8.0)^d^	.34	2 (1.6)		2 (2.9)^e^	0 (0)	>.99
Oxygen requirement	3 (2.5)	0 (0)	3 (6.0)	.14	0 (0)		0 (0)	0 (0)	N/A
Hospitalization	7 (5.8)	3 (4.3%)	4 (8.0)	.65	4 (3.3)		3 (4.3)	1 (2.0)	.86
Able to work^f^	10 (27.0)	8 (33.3)	2 (15.4)	.33	19 (51.4)		13 (54.2)	6 (46.2)	.57

^a^ICU: intensive care unit.

^b^N/A: not applicable.

^c^Gastrointestinal infection.

^d^2 patients with lower respiratory tract infections and 2 patients with urinary tract infections.

^e^1 patient with lower respiratory tract infection and 1 patient with gastrointestinal infection.

^f^Percentages are based on responses from 37 patients who were employed, including 24 at 2-week follow-up and 13 at 6-week follow-up.

Ongoing symptomatic COVID-19 was reported among 70% (35/50) and 54% (38/70) of the patients requiring ICU and non-ICU care, respectively, during their hospital stay (Table 3).

**Figure 4:**
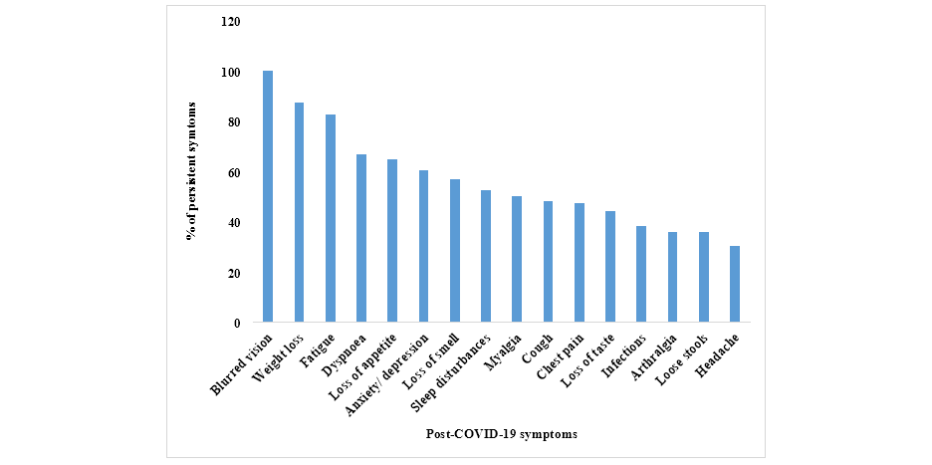
Persistence of post-COVID-19 symptoms in the sixth week of follow-up.

Comparison between post-COVID-19 symptoms of the non-ICU and ICU patients during the second week exhibited statistically significant differences in cough (*P*=.04) and weight loss (*P*=.003). At the time of the first follow-up, 2.5% (n=3) of patients had persistent hypoxia necessitating supplemental oxygen. Hospitalization due to postdischarge symptoms was reported in 11 patients (9.2%) at the 6-week follow-up. Among 37 patients in the whole cohort who were employed, 27% (n=10) and 51.4% (n=19) were able to rejoin work at 2 weeks and 6 weeks postdischarge from the hospital, respectively. Among the symptoms, the maximal reduction at 6 weeks was observed for fatigue at 11.7% compared to that experienced at the 2-week follow-up. Characteristics of post-COVID-19 symptoms that persisted at the 6th week of follow-up are depicted in Figure 4.

Association of baseline characteristics and COVID-19 treatment with the presence of symptoms at 6 weeks postdischarge is depicted in Table 4. Among patients discharged from the ICU, a higher proportion of females remained symptomatic at 6 weeks of follow-up as compared to males (odds ratio [OR] 8.27, 95% CI 0.97-70.2; *P*=.04). Among patients admitted in non-ICU locations, antiviral treatment (*P*=.007) and steroid administration (*P*=.001) during the hospital stay, and patients receiving steroids at discharge (*P*=.04) were significantly associated with the presence of post-COVID-19 symptoms at 6 weeks.

Female sex (OR 2.4, 95% CI 1.03–5.58; *P*=.04) and steroid administration the during hospital stay (OR 4.43, 95% CI 1.9-10.28; *P*=.001) were found to be significant risk factors for the presence of post-COVID-19 symptoms at 6 weeks as revealed by logistic regression analysis.

**Table 3:** Prevalence of post-COVID-19 symptoms during the follow-up period.

Symptom frequency		Overall (N=120)	Non-ICU^a^ patients (n=70)	ICU patients (n=50)
**Second week postdischarge (acute COVID-19), n (%)**				
	Presence of symptoms	94 (78.3)	49 (70.0)	45 (90.0)
	No symptoms	26 (21.7)	21 (30.0)	5 (10.0)
	One symptom	9 (7.5)	7 (10.0)	2 (4.0)
	Two symptoms	28 (23.3)	17 (24.3)	11 (22.0)
	More than two symptoms	57 (47.5)	25 (35.7)	32 (64.0)
**Sixth week postdischarge (ongoing symptomatic COVID-19), n (%)**				
	Presence of symptoms	73 (60.8)	38 (54.3)	35 (70.0)
	No symptoms	47 (39.2)	32 (45.7)	15 (30.0)
	One symptom	18 (15.0)	9 (12.9)	9 (18.0)
	Two symptoms	28 (23.3)	15 (21.4)	13 (26.0)
	More than two symptoms	27 (22.5)	14 (20.0)	13 (26.0)

^a^ICU: intensive care unit.

**Table 4:** Association of baseline characteristics and COVID-19 treatment with presence of symptoms at 6 weeks postdischarge.

Variables		Whole cohort (N=120)				ICU^a^ (n=50)				Non-ICU (n=70)		
		Symptoms (n=73)	No symptoms (n=47)	*P* value	Symptoms (n=35)		No symptoms (n=15)	*P* value	Symptoms (n=38)		No symptoms (n=32)	*P* value
Age (years), mean (SD)		56.71 (6.16)	52.21 (19.52)	.17	61.34 (12.79)		54.8 (13.49)	.11	52.45 (17.86)		51 (21.87)	.76
**Gender, n (%)**				.16				.04				.50
	Male	37 (56.1)	29 (43.9)		22 (61.1)		14 (38.9)		15 (50.0)		15 (50.0)	
	Female	36 (66.7)	18 (33.3)		13 (92.9)		1 (7.1)		23 (57.5)		17 (42.5)	
Charlson Comorbidity Index, mean (SD)		2.30 (1.91)	1.79 (2.04)	.16	2.74 (1.85)		1.87 (2.26)	.16	1.89 (1.90)		1.75 (1.96)	.76
**Antivirals, n (%)**												
	During hospital stay	55 (70.5)	23 (29.5)	.006	31 (70.5)		13 (29.5)	.51	24 (70.6)		10 (29.4)	.007
	During discharge	4 (57.1)	3 (42.9)	.57	2 (50.0)		2 (50.0)	.34	2 (66.7)		1 (33.3)	.50
**Anticoagulants, n (%)**												
	During hospital stay	51 (64.6)	28 (35.4)	.17	29 (69.0)		13 (31.0)	.54	22 (59.5)		15 (40.5)	.24
	During discharge	31 (70.5)	13 (29.5)	.07	20 (69.0)		9 (31.0)	.55	11 (73.3)		4 (26.7)	.08
**Steroids, n (%)**												
	During hospital stay	45 (75.0)	15 (25.0)	.001	33 (68.8)		15 (31.3)	.48	12 (100.0)		0 (0)	.001
	During discharge	16 (66.7)	8 (33.3)	.60	11 (57.9)		8 (42.1)	.12	5 (100.0)		0 (0)	.04

^a^ICU: intensive care unit.

## Discussion

### Principal Findings

Our study revealed a 61% incidence of post-COVID-19 syndrome at 6 weeks and the predominant symptoms included fatigue (55.8%), dyspnea (20%), and weight loss (16.7%). Importantly, the prevalence of these symptoms was not significantly different between those requiring critical care and those who were hospitalized but did not require intensive care. In addition, our study identified female sex and use of steroids during the hospital stay as significant predictors of persistent symptoms at 6 weeks.

As the media and public attention related to COVID-19 was primarily focused on mortality, the political machinery in the LMIC setting has been in a state of hubris to claim a low death rate; consequently, the morbidity due to post-COVID-19 syndrome, which is by large a societal health issue, remained overshadowed and unaddressed. There has been no systematic approach to capture the incidence and formulate treatment protocols for those who continue to suffer postinfective symptoms. Our study shows that a large proportion (61%) of survivors report persistent symptoms. As pointed out by others, this result suggests that the number reported here could be a significant underestimation on account of ascertainment bias, and the chance that others not requiring hospitalization or not able to access appropriate care during a peak wave could also suffer such consequences [8].

The predominant post-COVID-19 symptoms identified in our study included fatigue (55.8%), dyspnea (20%), and weight loss (16.7%), and 10% of the cohort reported other symptoms such as loss of appetite and cough. A UK-based study reported fatigue in 72% of ICU patients and in 60.3% of patients in other wards, and dyspnea in 65% of ICU patients and 42.6% of patients in other wards at 4-8 weeks after discharge [19]. Another prospective cohort study from the United States found fatigue and dyspnea reported among 55% and 45.3% of COVID-19 survivors at 35 days postdischarge, respectively [20]. Our study observations at 6 weeks postdischarge were overall in accordance with these previous publications regarding the prevalence of post-COVID-19 symptoms. However, our cohort had a lower prevalence of respiratory symptoms such as dyspnea and cough at 20% and 10%, respectively. The appearance of new post-COVID-19 symptoms at 2 weeks among the patients in our cohort included anxiety/depression, weight loss, sleep disturbance, blurred vision, skin rashes, and infections. Despite the possibility of postintensive care syndrome interfering with the post-COVID-19 syndrome assessment, the lack of a significant difference in the prevalence of post-COVID-19 symptoms at 6 weeks among ICU- and non-ICU–requiring patients in our study suggests that patients discharged from non-ICU locations with relatively lower COVID-19 severity could also develop post-COVID-19 symptoms similar to patients discharged from the ICU.

Despite the existing publications portraying the major role of comorbidities, advancing age, and LOS in the development of postdischarge COVID-19 symptoms, our data failed to show any significant association with these covariates [21]. In our study, female sex and steroid administration during the hospital stay were found to be significant risk factors for the persistence of post-COVID-19 symptoms at 6 weeks. Similar observations regarding female sex as a risk factor for developing post-COVID-19 symptoms was found in a prospective cohort study conducted by Mahmud et al [22] and in a retrospective multicenter study conducted by Zhang et al [23]. In addition, our study identified that patients who received steroids during their hospital stay (12/70, 17%) and at discharge (5/70, 7%) had post-COVID-19 symptoms at 6 weeks of follow-up irrespective of whether they had an ICU stay during hospitalization. Steroid-associated myopathy could have contributed to the fatigue-like state that persisted in some patients, since steroid use itself was associated with a higher chance of reporting this symptom [24]. This indicates a potential role for steroid stewardship programs that optimize the use of steroids in COVID-19 patients with special reference to mild cases not requiring intensive care, given the increased risk of lingering post-COVID-19 symptoms after discharge [25]. Therefore, patients receiving steroids discordantly should be under heightened surveillance for post-COVID-19 syndrome, particularly if they belong to the mild to moderate severity category.

The rate of hospitalization of patients after discharge from the hospital was 5.8% and 3.3% at the second and sixth week of follow-up, respectively. These rehospitalizations were due to the presence of post-COVID-19 symptoms; thus, any service targeting these subjects will have to distinguish among complaints as having a distinct pathophysiologic correlate to the acute infection, such as sequelae like lung fibrosis and ongoing inflammation related to lung damage, which would also have therapeutic implications. A retrospective study of hospitalized patients for post-COVID-19 sequelae demonstrated a higher risk of poor clinical outcomes, including mortality, as compared to that of the general population [26]. Richard Horton famously labeled the COVID-19 pandemic a “syndemic” [6]. Most notably, compared to other respiratory illnesses such as influenza, COVID-19 survivors were determined to suffer higher rates of rehospitalization and all-cause mortality in addition to mental health and cognitive impairment, posing a substantial burden on the health care system [27,28] owing to the syndemic nature of COVID-19 [6,29]. The concept of COVID-19 being a syndemic implies that it cannot be considered as simply an acute viral infectious syndrome but rather as a disease with long-lasting health effects, which requires the adoption of an interdisciplinary approach as a way forward for comprehensive management of this underrecognized health condition.

Our study also highlights higher risks faced by women in developing persistent post-COVID-19 symptoms. Unfortunately, women are known to be at the bottom heap in terms of health care availability and delivery in India [30], with poor accessibility to health-related services, particularly for symptoms such as fatigue, which is recognized to be widespread across many other postinfectious states and needs a comprehensive approach to its management [8].

In LMIC settings, where health expenditure is largely out of pocket [27,31], the majority of patients who suffer from post-COVID-19 syndrome—as an ill-defined clinical entity—are likely to ignore the health effects by not seeking the appropriate medical care. In this context, defining the illness and identifying its magnitude is of paramount importance, along with determining the predictive risk factors that would predispose an individual for this adverse health condition [32]. Our study identified women as having a higher risk of post-COVID-19 syndrome, which is a significant finding considering that in LMICs, neglected populations, including women and older adults, are also on the fringes of the health care delivery apparatus [33]. This study highlights the significant impact of COVID-19 on its survivors, pointing toward the need for holistic care for those who are identified as high risk and the unmet need for surveillance for post-COVID-19 issues.

Our study represents data from an unvaccinated population; thus, the realm of post-COVID-19 symptoms in the postvaccination era is worth capturing to elicit the possible difference brought about by vaccination. The reduced post-COVID-19 symptom burden among the vaccinated who suffer from breakthrough infections, as evidenced by the ZOE COVID Study, although promising, is in turn a call to address the existing global vaccine inequity [34] and to address the prevailing vaccine hesitancy.

In our plea for the recognition of the syndemic is a hope that we also recognize the need for a country-specific strategy to address the health needs of the majority population in the postpandemic era via harnessing the potential of technology. For example, this could be achieved by adopting and validating rapidly deployable strategies such as telemedicine to support post-COVID-19 symptoms such as fatigue, along with addressing the mental health issues, especially in the context of the persistent and looming threat of potential outbreaks from the newer and emerging variants. While these interventions have shown mixed results [35-37], the effects in post-COVID-19 syndrome recognition and management are still unexplored. Our study seeks to draw attention to the large group of hospitalized COVID-19 survivors who developed post-COVID-19 syndrome during the initial phase of the pandemic, especially women and patients treated with steroids who have an enhanced risk, irrespective of the COVID-19 severity. Future research is required to estimate the prevalence of persistent, recurrent, and new post-COVID-19 symptoms associated with COVID-19 infection and the changes in trends with vaccination and variants.

### Limitations

This study has many limitations. Our conclusion is based on data from a single tertiary-care center, which largely catered to morbidly ill patients. This study was limited to COVID-19 survivors who were hospitalized. Since the period of the follow-up was limited to 6 weeks, the duration of the persistence of the symptoms of long COVID beyond this period could not be determined. The prevalent SARS-CoV-2 variant during the study period was Delta; however, variant-specific influences on the incidence and severity of post-COVID-19 syndrome have not been investigated, which would require future prospective studies. Similarly, our study observations cannot be extrapolated to a vaccinated population as the study cohort was unvaccinated. However, this adds strength to the study as in the current context we are unlikely to obtain an unvaccinated cohort for studying the effects of long COVID. We did not use structured questionnaires to quantitatively measure the mood or fatigue given the lack of validation of most global instruments in our population.

### Conclusions

Our study revealed a high prevalence of post-COVID-19 symptoms at a minimum of 6 weeks postdischarge among inpatients who recovered from COVID-19. All severity categories of COVID-19 had an equal risk of contracting post-COVID-19 syndrome after hospitalization irrespective of their status of ICU or non-ICU stay. Female sex and steroid administration during the hospital stay were identified as predictors of the persistence of post-COVID-19 symptoms at 6 weeks. Health care systems across the globe have to account for the tail end crisis of the COVID-19 pandemic (ie, post-COVID-19 syndrome). Understanding the syndemic nature of the disease will help in formulating strategies for the management of COVID-19 by duly emphasizing the rehabilitation process of those who are suffering from an extended illness.

## Abbreviations

ICU: intensive care unit
LMIC: low- and middle-income country
LOS: length of hospital stay
OR: odds ratio

## Data Availability

The data sets used and/or analyzed during the current study are available from the corresponding author on reasonable request.
